# Epidemiology, Mode of Injury, and Treatment Outcomes in Neck Trauma: A Retrospective Study

**DOI:** 10.7759/cureus.110334

**Published:** 2026-06-06

**Authors:** Haneesha Bandari, S.M. Azeem Mohiyuddin, Sagayaraj A, Meenavalli Rohitha

**Affiliations:** 1 Department of Otorhinolaryngology - Head and Neck Surgery, Sri Devaraj Urs Medical College, Kolar, IND

**Keywords:** cut-throat injury, emergency tracheostomy, laryngotracheal injury, neck trauma, psychiatric morbidity

## Abstract

Objective: This study aimed to evaluate the epidemiological profile, injury patterns, anatomical involvement, management strategies, and clinical outcomes of patients presenting with neck trauma at a tertiary-care center. In addition, the study sought to identify demographic and clinical predictors of emergency airway intervention (tracheostomy) and psychiatric morbidity and to analyze their association with key clinical outcomes using appropriate statistical methods, including multivariate analysis.

Methodology: This retrospective observational study was conducted in the Department of Otorhinolaryngology and Head and Neck Surgery at a tertiary-care teaching hospital from January 2013 to March 2025. Patients of all ages presenting with blunt or penetrating neck trauma who underwent surgical exploration were included through consecutive sampling. Data regarding demographics, mechanism of injury, anatomical zone (Roon and Christensen classification), structural involvement, surgical procedures, and outcomes were retrieved from medical records. All patients were managed according to Advanced Trauma Life Support (ATLS) principles, with priority given to airway stabilization followed by appropriate surgical intervention.

Results: A total of 51 patients were included. Most were male (47, 92.2%), and 28 (54.9%) were older than 30 years. Suicidal injury was the most common mechanism (27, 52.9%), with sharp trauma reported in 39 (76.4%) cases. Zone II was the most frequently involved anatomical region (33, 64.7%). A tracheal breach was observed in 30 (58.8%) patients, a cricothyroid tear in 16 (31.3%), and a major vascular injury in 11 (21.5%). Emergency tracheostomy was required in 24 (47.1%) patients, whereas 16 (31.4%) underwent primary repair without tracheostomy. Successful decannulation was achieved in 23 (45.1%) patients, and only one (2%) patient required permanent tracheostomy. Psychiatric morbidity was identified in 23 (45.1%) patients. Most patients (37, 72.5%) had a hospital stay of 5-10 days.

Conclusion: Neck trauma predominantly affected young adult males and was most commonly caused by suicidal sharp injuries involving Zone II. Laryngotracheal injuries were frequent and often necessitated emergency tracheostomy; however, overall airway outcomes were favorable, with most patients successfully decannulated. Major vascular injuries occurred in a notable proportion of cases, requiring prompt surgical management. The high rate of postoperative psychiatric morbidity underscores the importance of integrating mental health support into trauma care to optimize overall outcomes.

## Introduction

Neck trauma accounts for 5-10% of major trauma presentations but carries high morbidity and mortality due to the close anatomical proximity of critical airway, vascular, neural, and aerodigestive structures. Even apparently superficial injuries may rapidly progress to airway compromise, major hemorrhage, or permanent functional deficits, necessitating immediate evaluation and intervention [1].

The epidemiology of neck trauma varies across populations and is influenced by socioeconomic conditions, behavioral factors, and regional violence patterns. Contemporary global and Indian data demonstrate an increasing proportion of penetrating injuries, particularly cut-throat wounds, frequently associated with interpersonal violence, alcohol use, and underlying psychiatric disorders [2,3]. Mathew et al. [1] reported a predominance of young adult males, with sharp-force trauma representing the principal mechanism of injury. Similar demographic patterns have been consistently observed in Indian cohorts, particularly among lower socioeconomic groups [2,3].

Historically, prior to the widespread use of firearms, penetrating neck trauma was predominantly caused by sharp weapons and edged instruments during interpersonal conflict, warfare, or self-inflicted injuries [3-10]. Interestingly, despite advances in modern trauma epidemiology, sharp-force injuries continue to constitute a major proportion of neck trauma cases in many developing regions, reflecting persistent sociocultural and behavioral influences [3].

The Roon and Christensen zonal classification remains fundamental for risk stratification and operative planning. Zone II, extending from the cricoid cartilage to the angle of the mandible, is more frequently involved due to its relative anatomical exposure and limited osseous protection [1,4]. Gupta et al. [5] reported predominant involvement of the mid-cervical region, with frequent extension into deeper aerodigestive structures.

Injuries involving the larynx, pharynx, or trachea constitute the most critical subgroup due to the risk of acute airway obstruction and aspiration. Clinical manifestations include dysphonia, subcutaneous emphysema, hemoptysis, and respiratory distress [3,4]. In the cohort reported by Gupta et al. [5], tracheal disruption occurred in more than one-third of patients, most of whom required emergency tracheostomy. Rajendiran et al. [6] documented laryngeal injury in 60% of cases and tracheostomy in 72% of cases, reflecting the severity of presentation in rural practice settings.

The mechanism of injury is strongly associated with underlying psychosocial determinants. Suicidal cut-throat injuries are commonly linked to psychiatric illness, family conflict, and alcohol dependence [2]. The Tamil Nadu study reported that over half of injuries were self-inflicted [6]. In contrast, Gupta et al. [5] identified homicidal assault as the predominant mechanism (56.7%). These findings highlight variability in injury patterns across regions and sociobehavioral factors.

Management requires adherence to Advanced Trauma Life Support (ATLS) principles, with airway stabilization as the highest priority. Early definitive airway control via endotracheal intubation or tracheostomy combined with hemorrhage control and layered anatomical repair is associated with improved outcomes and reduced complication rates [3,4]. Recent evidence supports a multidisciplinary approach involving otorhinolaryngology, trauma surgery, anesthesia, and psychiatric services, particularly in cases of self-harm [5,6].

The present study, conducted at a tertiary-care center in a rural region of a developing country, evaluates epidemiological characteristics, injury patterns, anatomical involvement, surgical management, and postoperative outcomes over a 12-year period. In addition, the study aims to identify demographic and clinical predictors of emergency airway intervention (tracheostomy) and psychiatric morbidity and to analyze their association with key clinical outcomes using appropriate statistical methods, including multivariate analysis. By systematically examining these factors, this study seeks to contribute to the evidence-based optimization of neck trauma management in resource-limited settings.

## Materials and methods

Study design and setting

This retrospective observational study was conducted in the Department of Otorhinolaryngology and Head and Neck Surgery at a tertiary-care teaching hospital in Kolar, Karnataka, India. The institution serves predominantly rural and semi-urban populations and manages emergency and surgical cases of neck trauma. As a record-based study, previously documented clinical and operative data were reviewed to analyze injury patterns, management strategies, and outcomes.

Study duration

Patients treated between January 2013 and March 2025 were included. The 12-year study period enabled adequate case accrual and assessment of temporal trends in injury patterns and management.

Ethical approval

The study was approved by the Institutional Ethics Committee (IEC No: SDUAHER/R&D/CEC/SDUMC-PG/124/NF/2025-26). Given the retrospective design, informed consent was waived. Patient confidentiality was maintained, and all identifying information was removed before analysis.

Study population

Patients of any age or sex presenting with blunt or penetrating neck trauma who underwent surgical exploration during the study period were included. Only surgically managed cases were analyzed to ensure accurate correlation between structural injury and operative findings.

Patients with isolated cervical spine injuries without airway or vascular involvement were excluded. Those with prior neck surgery were also excluded due to altered anatomy that could confound interpretation. Records with incomplete documentation were omitted from the analysis.

Sample size and sampling method

As this was a retrospective study, no prior sample size calculation was performed. All eligible patients meeting the inclusion criteria during the study period were included. Consecutive sampling was adopted to ensure comprehensive inclusion of surgically managed cases.

Data collection

Data were retrieved from electronic medical records and physical case files using a structured data collection form. Demographic variables included age, sex, and socioeconomic status. History of alcohol or tobacco use was recorded when available.

Clinical variables included mechanism of injury (suicidal, homicidal, or accidental), type of trauma (sharp or blunt), and anatomical zone based on the Roon and Christensen classification. Intraoperative findings were reviewed to document involvement of the trachea, cricothyroid membrane, hypopharynx, major vessels, and thyroid gland.

Airway status at presentation, the need for an emergency tracheostomy, surgical procedures performed, and details of vascular repair were documented. Postoperative variables included duration of intensive care unit (ICU) stay, total hospital stay, decannulation status, complications, and need for psychiatric referral or admission. Each case was assigned a unique study identification number to ensure anonymity.

Surgical management protocol

The primary surgical management of laryngotracheal and aerodigestive injuries was performed by the Department of Otorhinolaryngology and Head and Neck Surgery. Cases involving major vascular injury, extensive soft tissue trauma, or polytrauma were managed collaboratively with trauma surgeons, general surgeons, vascular surgeons, and anesthesiology teams as part of a multidisciplinary approach. All patients were managed according to ATLS principles, with airway stabilization as the primary priority. Indications for emergency airway intervention included respiratory distress, stridor, expanding cervical hematoma, visible laryngotracheal disruption, active hemorrhage, or altered sensorium.

**Figure 1:**
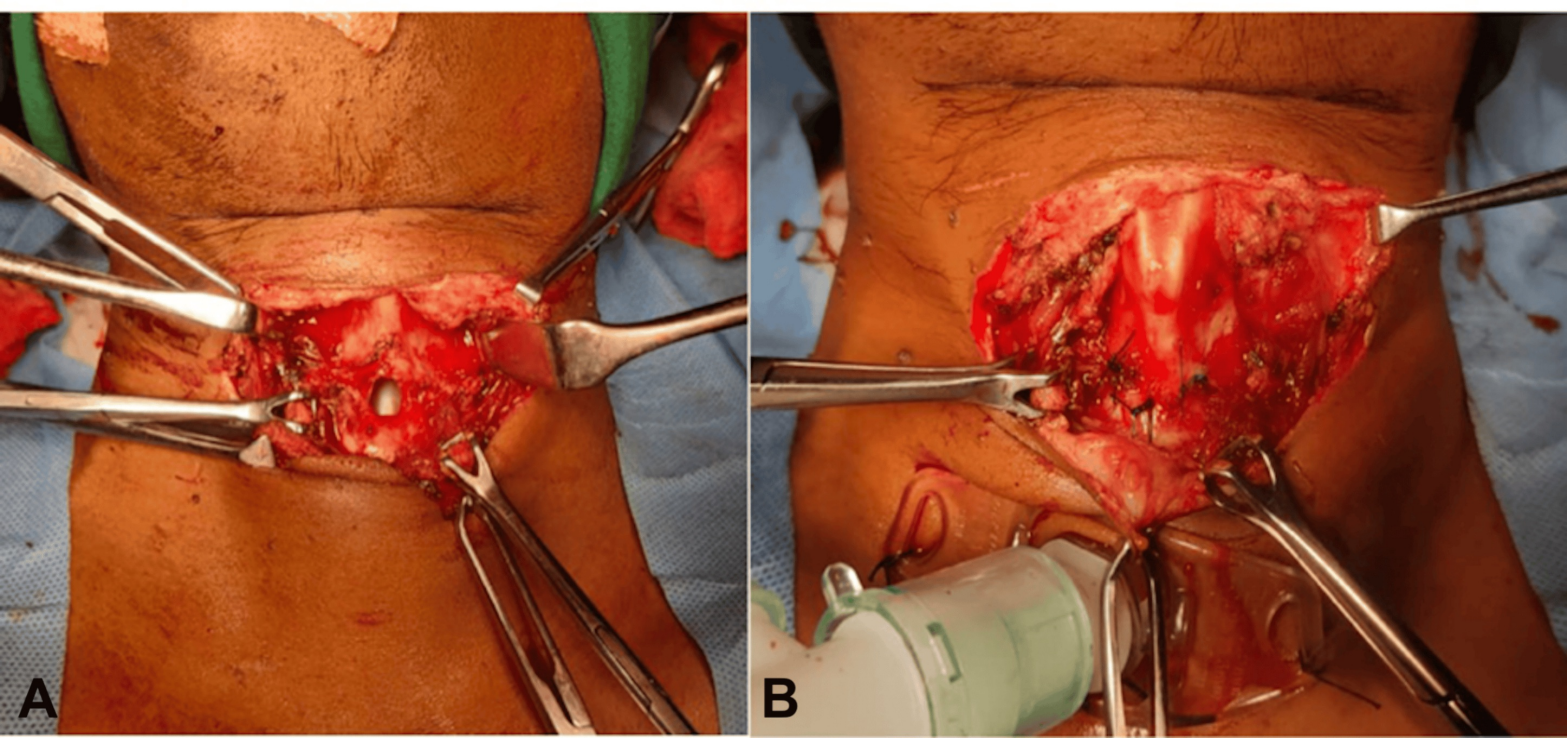
Intraoperative finding showing (A) cricothyroid membrane laceration in a self-inflicted cut-throat injury and (B) subsequent repair

In patients with airway compromise or deep laryngotracheal injury, emergency tracheostomy was performed under local or general anesthesia, depending on hemodynamic stability. In critically unstable patients, temporary endotracheal intubation through the open wound was performed in the emergency department before definitive surgical airway establishment (Figure 1). Definitive surgical exploration and repair procedures were subsequently performed in the operating room under sterile conditions following initial airway stabilization and hemodynamic optimization.

Surgical exploration involved extension of the existing wound along natural skin creases when feasible. Devitalized tissue was debrided, hematoma evacuated, and meticulous hemostasis achieved. Injuries to the cricothyroid membrane and trachea were repaired primarily using interrupted absorbable sutures with careful mucosal approximation to minimize the risk of postoperative stenosis. Laryngeal framework injuries were assessed for cartilage disruption and repaired as indicated. Hypopharyngeal injuries were repaired in layers with absorbable sutures to ensure watertight closure. A nasogastric tube was placed to facilitate enteral feeding and prevent salivary contamination during healing.

Major vascular injuries were managed with primary repair or ligation, depending on vessel type, extent of damage, and patient stability. Venous injuries were typically treated with ligation, whereas arterial injuries were repaired whenever feasible. Thyroid gland injuries were managed with hemostatic suturing or partial lobectomy when required. Wounds were closed in layers over drains when indicated. Closed suction drains were placed selectively in patients with extensive soft tissue dissection, hypopharyngeal repair, vascular injury, significant dead space, or anticipated postoperative fluid collection. Smaller uncomplicated wounds without significant contamination, dead space, or persistent oozing were managed without drain placement. Broad-spectrum intravenous antibiotics were administered perioperatively. Broad-spectrum intravenous antibiotics were administered perioperatively. Postoperatively, patients were monitored in the ICU based on airway status and hemodynamic stability. Tracheostomized patients underwent regular airway assessment and wound evaluation. Gradual decannulation trials were initiated once airway edema subsided and adequate laryngotracheal healing was confirmed clinically.

Outcome measures

The primary outcomes were airway outcomes (successful decannulation or the need for a permanent tracheostomy) and hospital stay duration. Secondary outcomes included injury pattern, structural involvement, surgical management type, and postoperative psychiatric morbidity.

Statistical analysis

Data were entered into Microsoft Excel (Microsoft Corporation, Redmond, Washington) and analyzed using IBM SPSS Statistics for Windows, Version 22 (Released 2013; IBM Corp., Armonk, New York). All variables were categorical and summarized using frequencies and percentages. Descriptive analysis was performed for demographic and clinical variables.

To identify factors associated with key outcomes, multivariate analysis was performed using binary logistic regression. Independent predictors of emergency tracheostomy and psychiatric morbidity were evaluated, and adjusted odds ratios (OR) with 95% confidence intervals (CI) were calculated. A p-value of <0.05 was considered statistically significant.

## Results

A total of 51 participants were included in the study. More than half of the patients were aged >30 years (28, 54.9%), whereas 23 (45.1%) were ≤30 years. The study population was predominantly male, comprising 47 (92.2%) participants, while females accounted for 4 (7.8%). A history of addictions was reported in 26 (51.0%) patients, whereas 25 (49.0%) had no addictions. Regarding socioeconomic status, the majority of participants (31, 60.7%) belonged to the lower socioeconomic group, while 20 (39.3%) were categorized in other socioeconomic classes (Table 1).

**Table 1:** Demographic characteristics of the study population

Variable	Category	Total number (N)	Percentage (%)
Age	≤30 years	23	45.1
	>30 years	28	54.9
Sex	Male	47	92.2
	Female	4	7.8
Addictions	Present	26	51.0
	Absent	25	49.0
Socioeconomic status	Lower	31	60.7
	Others	20	39.3

Suicidal injuries were present in 27 (52.9%) patients, followed by homicidal injuries in 15 (29.4%) and accidental injuries in 9 (17.6%) patients. Sharp trauma was observed in 39 (76.4%) patients, whereas blunt trauma was seen in 12 (23.6%) patients. Injuries involving Zone II were identified in 33 (64.7%) patients, while combined Zone I and Zone II injuries were present in 18 (35.3%) patients (Table 2).

**Table 2:** Manner, type, and zone of injury among patients with neck trauma

Variable	Category	Frequency (n)	Percentage (%)
Manner of injury	Suicidal	27	52.9
	Homicidal	15	29.4
	Accidental	9	17.6
Type of trauma	Sharp	39	76.4
	Blunt	12	23.6
Zone of injury	Zone II	33	64.7
	Zone I + II	18	35.3

Tracheal breach was the most frequently involved structure, observed in 30 (58.8%) patients, indicating that airway compromise was a common finding in neck trauma cases. Cricothyroid membrane tear was the second most common injury, present in 16 (31.3%) patients. Major vascular injuries were identified in 11 (21.5%) cases, reflecting a considerable proportion of potentially life-threatening presentations. Hypopharyngeal involvement was noted in 8 (15.6%) patients, suggesting that aerodigestive tract injury was associated in a subset of cases. Thyroid gland injury was relatively uncommon, occurring in only 4 (7.8%) patients (Table 3).

**Table 3:** Structures involved in neck trauma cases Frequencies exceed the total sample size because individual patients sustained injuries to multiple anatomical structures.

Structure involved	Frequency (n)	Percentage (%)
Tracheal breach	30	58.8
Cricothyroid membrane tear	16	31.3
Major vessel injury	11	21.5
Hypopharyngeal injury	8	15.6
Thyroid gland injury	4	7.8

Emergency tracheostomy, combined with wound exploration and repair, was the most commonly performed intervention, performed in 24 (47.1%) patients, highlighting the high proportion of cases requiring immediate airway stabilization. Wound exploration with primary repair without tracheostomy was performed in 16 (31.4%) patients, indicating that nearly one-third of cases were managed without a definitive surgical airway. Major vascular repair or ligation was required in 11 (21.5%) patients, reflecting the significant number of patients presenting with vascular involvement requiring urgent surgical management (Table 4).

**Table 4:** Management modalities adopted in neck trauma patients

Management	Frequency (n)	Percentage (%)
Emergency tracheostomy + wound exploration/repair	24	47.1
Wound exploration + primary repair (without tracheostomy)	16	31.4
Major vascular repair/ligation	11	21.5

Successful decannulation was achieved in 23 (45.1%) patients, indicating favorable airway recovery in the majority of cases requiring tracheostomy, whereas only 1 (2.0%) patient required a permanent tracheostomy. Psychiatric ward admission or documented psychiatric issues were noted in 23 (45.1%) patients, reflecting a substantial psychological component associated with the injuries. Regarding hospital stay duration, most patients (37, 72.6%) had a stay of 5-10 days, while 10 (19.6%) were discharged within 5 days. A prolonged hospital stay of more than 10 days was observed in 4 (7.8%) patients (Table 5).

**Table 5:** Airway, psychiatric, and hospital stay outcomes among patients

Outcome	Category	Frequency (n)	Percentage (%)
Airway outcome	Successfully decannulated	23	45.1
	Permanent tracheostomy	1	2.0
Psychiatric outcome	Psychiatry ward admission/psychiatric issues	23	45.1
ICU/hospital stay duration	<5 days	10	19.6
	5–10 days	37	72.6
	>10 days	4	7.8

On multivariate logistic regression analysis, tracheal breach (OR: 5.82, p=0.003) and cricothyroid membrane tear (OR: 4.21, p=0.017) were identified as independent predictors of emergency tracheostomy. Other demographic and injury-related variables did not show a statistically significant association (Table 6).

**Table 6:** Multivariate logistic regression analysis for predictors of emergency tracheostomy * shows a significant p-value

Variable	Adjusted odds ratios	95% confidence interval	p-value
Age > 30 years	1.42	0.48–4.18	0.52
Male sex	1.76	0.15–20.2	0.65
Suicidal injury	1.89	0.64–5.54	0.24
Sharp trauma	2.31	0.71–7.48	0.16
Zone II injury	1.58	0.52–4.76	0.41
Tracheal breach	5.82	1.78–19.01	0.003*
Cricothyroid membrane	4.21	1.29–13.68	0.017*
Major vascular injury	2.07	0.59–7.21	0.25

Psychiatric morbidity was independently associated with suicidal mode of injury (OR: 6.74, p=0.002) and history of addictions (OR: 3.95, p=0.016) (Table 7).

**Table 7:** Multivariate logistic regression analysis for predictors of psychiatric morbidity * shows a significant p-value

Variable	Adjusted odds ratios	95% confidence interval	p-value
Age > 30 years	1.36	0.46–3.98	0.58
Male sex	1.22	0.10–14.5	0.87
Suicidal injury	6.74	2.01–22.6	0.002*
Addictions	3.95	1.28–12.18	0.016*
Sharp trauma	1.88	0.55–6.3	0.31

Patients who underwent tracheostomy had approximately 3.9 times higher odds of prolonged hospitalization (AOR = 3.88, 95% CI: 1.12-13.4, p = 0.032). Similarly, the presence of vascular injury was associated with a 4.6-fold increase in the likelihood of extended hospital stay (AOR = 4.56, 95% CI: 1.21-17.1, p = 0.025). Although hypopharyngeal injury was associated with increased odds (AOR = 3.12), the association was not statistically significant (95% CI: 0.88-11.0, p = 0.08). These findings suggest that tracheostomy and vascular injury are important predictors of prolonged hospitalization, while hypopharyngeal injury may have a potential but non-significant association (Table 8).

**Table 8:** Predictors of prolonged hospital stay (>10 days) * shows a significant p-value

Variable	Adjusted odds ratios	95% confidence interval	p-value
Tracheostomy	3.88	1.12–13.4	0.032*
Vascular injury	4.56	1.21–17.1	0.025*
Hypopharyngeal injury	3.12	0.88–11.0	0.08

**Figure 2:**
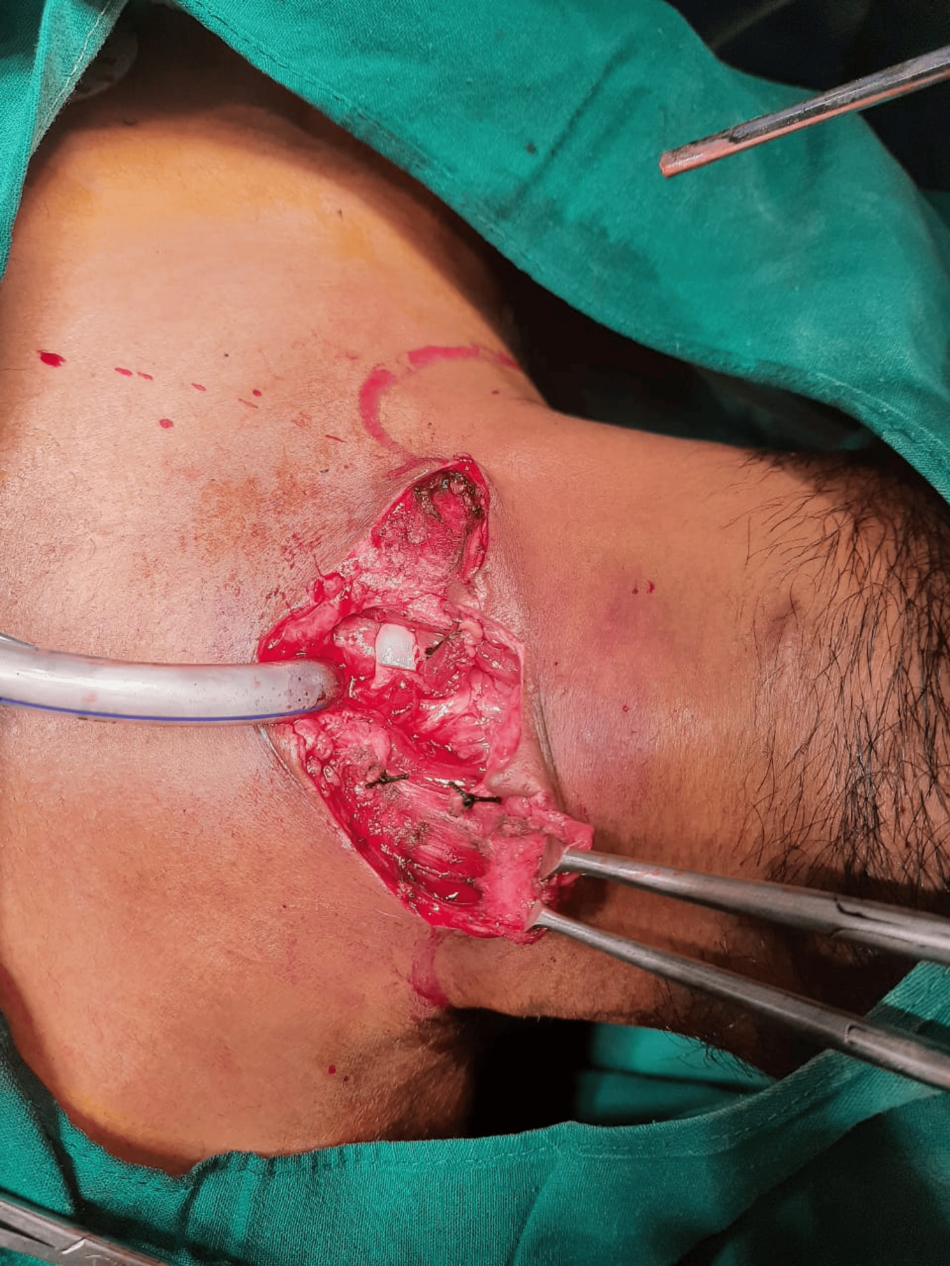
Image showing endotracheal tube insertion through neck laceration by the emergency unit

Intraoperatively, a tracheal breach was confirmed in 30 (58.8%) patients, many of whom required formal tracheal repair in addition to airway diversion. Cricothyroid membrane tears in 16 (31.3%) patients were repaired with layered closure to restore laryngeal integrity (Figure 1). Hypopharyngeal injuries were observed in 8 (15.6%) patients and were closed primarily in two layers with placement of nasogastric feeding tubes. Major vascular injuries occurred in 11 (21.5%) patients and required either primary repair or ligation to achieve hemorrhage control. Thyroid gland injuries were documented in 4 (7.8%) patients and were managed conservatively with hemostatic suturing or limited resection. Temporary endotracheal intubation via the neck wound was performed in selected emergency cases before definitive tracheostomy (Figure 2), reflecting the severity of airway disruption at presentation.

## Discussion

Traumatic neck injuries remain a complex clinical challenge due to the proximity of critical airway, vascular, and neural structures and the risk of rapid airway compromise. In the present study, there was a marked male predominance (>90%), consistent with multiple published studies reporting a higher incidence among males, likely related to greater exposure to interpersonal violence and high-risk behaviors. A large epidemiologic review from a Level I trauma center similarly reported male predominance in traumatic neck injuries, underscoring the influence of demographic factors on injury patterns [7]. Singhai et al. also reported a male predominance in ENT trauma patients and attributed this to greater outdoor activity and higher risk exposure among males [8].

The mean age in our cohort was above 30 years, indicating the predominant involvement of the economically productive age group. This finding aligns with Sachdeva and Vatsyayan [2], who reported a mean age of approximately 27 years in a prospective Indian cohort of laryngotracheal injuries, with tracheal breach observed in 79% of cases. Comparable age distribution and airway involvement were reported in another observational study of penetrating neck trauma, highlighting increased vulnerability among younger and middle-aged adults [9].

Regarding the mechanism, suicidal injuries constituted a higher proportion in our cohort compared with homicidal or accidental causes. Mahmoodi et al. [10], in a retrospective review of 192 penetrating neck trauma cases, reported stab wounds with predominantly homicidal intent as the most frequent mechanism. In contrast, our findings likely reflect regional and sociocultural influences contributing to a greater proportion of self-inflicted injuries. Population-based data from Al-Thani et al. [11] identified motor vehicle collisions as the leading cause of blunt neck trauma in their region, further illustrating geographic variation in trauma epidemiology. Unlike several Western trauma series where blunt trauma secondary to road traffic accidents predominates, our cohort demonstrated a higher proportion of penetrating sharp injuries, likely reflecting regional sociocultural factors and the high prevalence of self-inflicted cut-throat injuries.

Zone II was the most frequently involved anatomical region in our series, consistent with findings by Rincon and Spataro [12], who identified Zone II as the most commonly injured zone due to its exposure and limited bony protection. Puttamadaiah et al. [9] similarly emphasized that penetrating Zone II injuries often require urgent airway control and surgical intervention because of the concentration of aerodigestive and vascular structures.

Airway compromise was a major finding in our study, with tracheal breach in more than half of the cases and frequent involvement of the cricothyroid membrane. These observations are consistent with Sachdeva and Vatsyayan [2], who reported tracheal injury as the predominant airway lesion. Rincon and Spataro [12], in a contemporary review, emphasized that early airway control, whether by endotracheal intubation or a surgical airway, is critical for reducing morbidity and mortality, underscoring the importance of prompt airway stabilization.

Vascular injury was observed in approximately 21% of patients, comparable to reports indicating vascular involvement in up to one-quarter of penetrating neck trauma cases. Such injuries significantly complicate management and increase the risk of hemorrhage and adverse outcomes. Mahmoodi et al. [10] also reported frequent vascular repair or ligation in their series.

Recent institutional experiences further support our findings. The Western Rajasthan study highlighted the complexity of laryngotracheal trauma and emphasized the need for early airway securing and definitive repair [13]. During the COVID-19 pandemic, altered healthcare access and delayed presentation influenced airway management, and a study from CMC Vellore reinforced the use of airway management protocols even under resource constraints [14]. These data consistently demonstrate that early airway assessment remains central to survival in cervical trauma.

Emergency tracheostomy was required in nearly half of our patients, comparable to other reports where tracheostomy or alternative surgical airway procedures were frequently necessary in cases of significant laryngeal or tracheal disruption [7]. In our cohort, 24 patients underwent tracheostomy; 23 were successfully decannulated (95.8%), and 1 required a permanent tracheostomy (4.2%) due to subglottic stenosis. When calculated for the entire cohort (n = 51), successful decannulation occurred in 45.1% (23/51), while permanent tracheostomy was required in 2.0% (1/51). These outcomes indicate favorable airway rehabilitation with timely surgical intervention and structured postoperative care, consistent with established principles in laryngotracheal trauma management [4,13,14]. The relatively low rate of permanent tracheostomy in our cohort may reflect timely airway stabilization, early definitive surgical repair, meticulous layered reconstruction, and careful postoperative airway surveillance, which together contributed to favorable long-term airway outcomes. In contrast, in broader laryngeal trauma cohorts, the reported rate of tracheostomy varied, but the importance of securing a definitive airway was consistently emphasized for patients with severe injury [15]. High rates of successful decannulation following acute airway injury management have also been reported in contemporary literature [16].

Most patients required hospital stays of 5-10 days, reflecting the need for airway monitoring, wound care, and rehabilitation. Notably, 45.1% of patients required psychiatric evaluation or admission postoperatively. This finding supports existing evidence that self-inflicted neck injuries are frequently associated with psychiatric illness, emotional distress, and substance dependence [2,6]. While surgical repair is essential for survival, psychiatric intervention is critical for preventing recurrence and supporting long-term recovery. These findings highlight the importance of integrated trauma care pathways involving otorhinolaryngologists, anesthesiologists, trauma surgeons, and mental health professionals.

Limitations

This study has several limitations that should be acknowledged. First, the retrospective design limited the availability of standardized clinical documentation, long-term follow-up data, and detailed functional outcome assessment. A prospective study design may have allowed more comprehensive evaluation of airway, voice, swallowing, and psychological outcomes. However, due to the emergent nature of neck trauma and the relatively low incidence of surgically managed cases, prospective randomization or controlled study design would have been difficult to implement ethically and practically.

Second, the study was conducted at a single tertiary-care institution with a relatively modest sample size (n = 51), which may limit the generalizability of the findings to other populations and trauma systems. The limited sample size may also have reduced the statistical power of subgroup analyses and multivariate regression models, increasing the possibility of a Type II error and limiting the detection of smaller associations.

Third, only surgically managed neck trauma cases were included in the present analysis. Patients with isolated cervical spine injuries without airway or vascular involvement and those managed conservatively were excluded, which may have influenced the observed epidemiological and clinical patterns.

In addition, although multivariate analysis identified tracheal breach and cricothyroid membrane injury as predictors of emergency tracheostomy, the stronger association observed with tracheal breach likely reflects the greater degree of airway disruption and higher risk of respiratory compromise associated with direct tracheal injury compared with isolated cricothyroid membrane tears.

Finally, although psychiatric morbidity was identified in a substantial proportion of patients, the study could not comprehensively evaluate long-term psychiatric outcomes or adherence to mental health follow-up due to the retrospective design. Larger multicenter prospective studies are required to evaluate injury mechanisms further, optimize multidisciplinary treatment strategies, and improve the integration of psychiatric care into trauma management pathways.

## Conclusions

Neck trauma constitutes a life-threatening emergency due to the proximity of critical airway and vascular structures. In this 12-year retrospective study, sharp injuries and Zone II involvement predominated, with frequent laryngotracheal injuries necessitating emergency tracheostomy and definitive surgical repair. Airway outcomes were favorable, with most tracheostomized patients successfully decannulated and only one requiring a permanent tracheostomy. The substantial proportion of patients requiring postoperative psychiatric intervention underscores the importance of incorporating structured mental health assessment and follow-up into standard trauma care pathways. Early airway stabilization, timely surgical management, and coordinated multidisciplinary care remain essential for reducing morbidity and optimizing outcomes in neck trauma.
